# Supplementary material for: Comparative Analysis of Saccharification Characteristics of Different Type Sweetpotato Cultivars
Source: Foods. 2023 Oct 15;12(20):3785. doi: 10.3390/foods12203785 (PMC10606501; doi:10.3390/foods12203785)
Supplement: Supplementary file 1 [file foods-12-03785-s001.zip › foods-2605442-supplementary.pdf]

Supplementary Table S1 Primers used in real-time PCR

Gene name	Sequence	Gene ID
IbAMY	ATTCTTCAGGCTGCTGTGCT	KT281131.1
	CTCCGGGATGGGTGAGGATA	
IbBAM	CGGGAAGAGACGGTTATCGG	D12882.1
	TGCATTCTCACCTGCCACAT	
IbSP	GAGGTGGCTGAAGATTGGCT	L25626.2
	CCCACCAATCCAGTGCTTCT	
IbSS	ATTCCCTGGACACCTTGCAG	AF068834.1
	TGCACATCTTGGCCCATCAT	
IbSBE	CGCCTTCCTGATGGTTCAGT	AB042937.1
	GTACTGGTGTTCTGCCGTCA	
IbPUL	GCTGCTCGACGATGCCTCT	
	CATCCTCAACGTCCACATTCC	
IbISAI	GGAACGAGGTGGTTATCGGTG	DQ074643
	TCTGGGCATAGCAACAGAATTATG	
IbPGM	TTCTGCTGGTGCAACAGTAAGAG	
	AGTTGGCTTCTCTCTTCCGGTA	
IbSSII	AGACTGTGGGATCTACTGAAAGGC	AF068834
	GTGAATCCACGTCCAGTGGC	
IbSSIII	TCTGTTATCCTGAGGAGGTAAAACC	
	CTCCCATGATCAATACATCAGGC	
IbSSIV	CTGCTTTCTCATTTCTGTCATCGT	
	GCTCAACTTCCACTTGACTCAGAG	
IbAGPase-TL1	GAGATATCCCACATCCAACGACTT	AJ252316.1
	TAGGGCCAAGTTAGCGTCGTAG	
IbAGPase-sTL1	AGAGAATTGACGGTGATGTTAGCA	Z79635.1
	ATGAACGGAGCAGTCCGAAC	
IbAGPase-sTL2	CCAAAAGGAGAACAGTTGAAAGCTA	Z79636.1
	CTCCAGGGAACTTTTCTCGAAGTA	
IbSBEI	ATTCTTGGCCTAGACCAAGGG	AB194725
	ACAATGCAGCCTTCTTCTTTGTTA	
IbSBEII	AGTCCGCTGTTTGGAGGCTT	AB071286
	CCTCAACTGGTTTTGCTTCGTC	
IbGBSSI	GACTGCGGCATCACTGGTATTT	AB071604
	GAACTTAGAAATCGCAGCAT	
IbARF	CTTTGCCAAGAAGGAGATGC	JX177359
	TCTTGTCCTGACCACCAACA	
